# Inhalable Fucoidan Microparticles Combining Two Antitubercular Drugs with Potential Application in Pulmonary Tuberculosis Therapy

**DOI:** 10.3390/polym10060636

**Published:** 2018-06-08

**Authors:** Ludmylla Cunha, Susana Rodrigues, Ana M. Rosa da Costa, M Leonor Faleiro, Francesca Buttini, Ana Grenha

**Affiliations:** 1 Centre for Biomedical Research, University of Algarve, 8005-139 Faro, Portugal; ludmyllacc@gmail.com (L.C.); susananasus@gmail.com (S.R.); mfaleiro@ualg.pt (M.L.F.); 2 Centre for Marine Sciences, University of Algarve, 8005-139 Faro, Portugal; 3 Algarve Chemistry Research Centre and Department of Chemistry and Pharmacy, University of Algarve, 8005-139 Faro, Portugal; amcosta@ualg.pt; 4 Food and Drug Department, University of Parma, 43124 Parma, Italy; francesca.buttini@unipr.it

**Keywords:** alveolar macrophages, fucoidan, isoniazid, inhalable microparticles, rifabutin, spray-drying, tuberculosis therapy

## Abstract

The pulmonary delivery of antitubercular drugs is a promising approach to treat lung tuberculosis. This strategy not only allows targeting the infected organ instantly, it can also reduce the systemic adverse effects of the antibiotics. In light of that, this work aimed at producing fucoidan-based inhalable microparticles that are able to associate a combination of two first-line antitubercular drugs in a single formulation. Fucoidan is a polysaccharide composed of chemical units that have been reported to be specifically recognised by alveolar macrophages (the hosts of *Mycobacterium*). Inhalable fucoidan microparticles were successfully produced, effectively associating isoniazid (97%) and rifabutin (95%) simultaneously. Furthermore, the produced microparticles presented adequate aerodynamic properties for pulmonary delivery with potential to reach the respiratory zone, with a mass median aerodynamic diameter (MMAD) between 3.6–3.9 µm. The formulation evidenced no cytotoxic effects on lung epithelial cells (A549), although mild toxicity was observed on macrophage-differentiated THP-1 cells at the highest tested concentration (1 mg/mL). Fucoidan microparticles also exhibited a propensity to be captured by macrophages in a dose-dependent manner, as well as an ability to activate the target cells. Furthermore, drug-loaded microparticles effectively inhibited mycobacterial growth in vitro. Thus, the produced fucoidan microparticles are considered to hold potential as pulmonary delivery systems for the treatment of tuberculosis.

## 1. Introduction

Tuberculosis (TB) is a leading infectious cause of death worldwide, even though a vaccine and several effective antibiotics are available for its prevention and treatment. According to the World Health Organisation (WHO), there were 10.4 million new TB cases globally and 1.3 million TB-related deaths in 2016 [1]. Global TB control is very difficult due to many factors, including late diagnosis and patient nonadherence to long-term treatments, which leads to a high incidence of extensive resistance to effective antitubercular drugs [2]. Besides drug resistance, the current therapy faces serious challenges, such as multi-drug interactions—especially with antiretroviral agents in cases of TB and HIV *co*-infection—long-term treatment, and antibiotic toxicity, which lead to adverse effects, resulting in patient non-compliance [3]. In this sense, accurate and early diagnosis are essential in TB therapy, in addition to patient adherence to the treatment and re-examination to verify the development of the active form of the disease. Moreover, in the treatment of TB, it must be considered the ability of *Mycobacterium tuberculosis* to survive intracellularly in the host alveolar macrophage for a long period. For that reason, therapy should ideally involve the intracellular delivery of antitubercular agents [4]. This could be achieved by the design of inhalable drug formulations with suitable aerodynamic properties to reach the alveoli, where macrophages infected with *M. tuberculosis* reside [5]. This strategy would potentially reduce the dosage and frequency of administration, and perhaps shorten treatment duration. As a result, systemic side effects could be avoided, improving patient adherence to the treatment [6]. 

**Figure 1:**
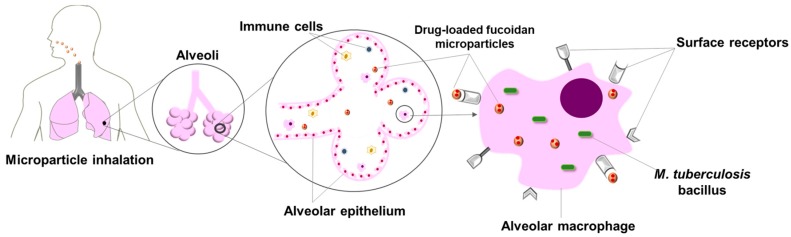
Illustration of microparticle uptake by alveolar macrophages, assuming targeted drug delivery mediated by fucoidan. Drug-loaded fucoidan microparticles reach the alveoli upon dry powder aerosolisation. Next, alveolar macrophages, infected with *M. tuberculosis*, engulf the microparticles. Fucoidan is expected to facilitate phagocytosis, because it possesses chemical moieties that are recognisable by the macrophage surface receptors.

In this context, fucoidan (FUC) could be used as the matrix material of the inhalable carriers to be designed. FUC is a promising biomaterial in this regard, because it presents sulphated fucose and other sugar residues [7], which can be recognised by the surface receptors of alveolar macrophages [8]. This can favour the internalisation of microparticles by alveolar macrophages, and subsequently, the delivery of drugs at the infection site. Furthermore, FUC can possibly intensify macrophage activation mediated by membrane receptors [9,10,11]. The working mechanism herein proposed for FUC microparticles is illustrated in Figure 1.

The purpose of this work is to produce inhalable FUC-based microparticles combining both isoniazid (INH) and rifabutin (RFB) in a single formulation, with a potential affinity for alveolar macrophages mediated by FUC. The proposed combined therapy of INH and RFB complies with the specific WHO guidelines on TB therapy [12]. The microparticles were characterised and their respirability was evaluated to determine the ability to reach the deep lung. The uptake of FUC microparticles by macrophages was assessed by flow cytometry, and their capacity to activate macrophages was determined. The potential antimicrobial activity of the produced carriers was also evaluated, along with the determination of their cytocompatibility.

## 2. Materials and Methods

### 2.1. Materials

Fucoidan (FUC, *Laminaria japonica*) and rifabutin (RFB, *M*_w_ 847.00 g/mol) were purchased from Chemos GmbH (Regenstauf, Germany). Isoniazid (INH, *M*_w_ 137.14 g/mol), buffer solution pH 5 (citric acid ~0.096 M, sodium hydroxide ~0.20 M), dimethylformamide (DMF), Dulbecco’s modified Eagle’s medium (DMEM), lipopolysaccharide (LPS), *N*-(3-dimethylaminopropyl)-*N*′-ethylcarbodiimide hydrochloride (EDAC), non-essential amino acids solution and penicillin/streptomycin (10,000 units/mL, 10,000 g/mL), sodium dodecyl sulphate (SDS), Triton-X 100, trypsin-EDTA solution (2.5 g/L trypsin, 0.5 g/L EDTA), trypan blue solution (0.4%), and HCl were supplied by Sigma-Aldrich (Munich, Germany). Thiazolyl blue tetrazolium bromide (MTT), phosphate buffer saline (PBS) tablets pH 7.4 and Tween 80^®^ were supplied by Amresco (Solon, OH, USA). Dimethyl sulfoxide (DMSO) was provided by VWR (Fontenay-sous-Bois, France) and phorbol 12-myristate 13-acetate (PMA) was provided by Cayman Chemicals (Ann Arbor, MI, USA). A lactate dehydrogenase (LDH) kit was obtained from Takara Bio (Tokyo, Japan) and l-glutamine solution (200 mM), as well as fetal bovine serum (FBS) from Gibco (Life Technologies, Waltham, MA, USA). RPMI 1640 and Ham’s F12 media were supplied by Lonza Group AG (Basel, Switzerland). Middlebrook 7H9 (M7H9; 4.7 g/L) and OADC (oleic acid, albumin, dextrose and catalase) were purchased from Remel (Lenexa, KS, USA). Ultrapure water (MilliQ, Millipore, UK) was used throughout the studies and other chemicals were reagent grade.

### 2.2. Preparation of Microparticles

Microparticles with and without drugs were obtained from 2% (*w*/*v*) FUC solutions, which were prepared by dissolving the polymer in ultrapure water under stirring (MS-3000 Biosan, Riga, Latvia). The drugs INH and RFB were incorporated into the solution in two steps. INH was ground in a porcelain mortar, solubilised in water and then added dropwise to the prepared polymeric solution. RFB was ground in a glass mortar, dissolved in 4% (*v/v*) ethanol, and then incorporated drop by drop into the polymeric solution containing INH. The final solution (50 mL) was stirred for 1 h before spray-drying. Drugs were included in the formulation to obtain FUC/INH/RFB mass ratios of 10/1/0.5. 

Microparticles were produced on a laboratory scale spray-dryer (Büchi B-290 Mini Spray Dryer, Büchi Labortechnik AG, Flawil, Switzerland), equipped with a high-performance cyclone. The equipment operated in open mode configuration, using compressed air. The spray flow rate was set at 473 L/h, and the operating parameters were set as indicated in Table 1.

The production yield of the spray-drying process was calculated comparing the weight of collected microparticles and the total amount of solids initially added to produce microparticles. Dry powders were stored in desiccators until further use.

Fluorescent microparticles of FUC were also produced to be used in the assay of microparticle uptake by macrophages. To produce fluorescently labelled FUC, the covalent attachment of the dye to the polymer was carried out by reacting it with fluorescein sodium salt in the presence of *N*-(3-dimethylaminopropyl)-*N*’-ethylcarbodiimide hydrochloride (EDAC) as activator of the fluorescein carboxyl group. In short, 1 g of FUC was dissolved at 2% (*w*/*v*) in water. Fluorescein sodium salt (24.4 mg) dissolved in 4 mL of 96% (*v/v*) ethanol and EDAC (9.6 mg dissolved in 16 mL of milli-Q water) were added to the former solution. The reaction mixture was kept under stirring in the dark overnight and afterwards dialysed (2000 Da *M*_w_ cut-off) against distilled water, which was also protected from the light. The dialysate was frozen and freeze-dried (FreeZone Benchtop Freeze Dry System, Labconco, Kansas City, MO, USA). Fluorescent (unloaded) FUC microparticles were prepared under the same conditions as displayed in Table 1.

### 2.3. Microparticle Characterisation

#### 2.3.1. Morphology

Microparticle morphology was visualised by field emission scanning electron microscopy (FESEM Ultra Plus, Zeiss, Jena, Germany). Briefly, the dry powder was placed onto metal plates and 5-nm thick iridium film was sputter-coated (model Q150T S/E/ES, Quorum Technologies, Lewes, UK) on the sample before visualisation.

#### 2.3.2. Feret’s Diameter

The microparticle size was estimated as the Feret’s diameter and measured as the mean of 300 microparticles (*n* = 3). The measurements were performed by optical microscopy (Microscope TR 500, VWR international, Leuven, Belgium).

#### 2.3.3. Particle Size Distribution

Drug-loaded FUC microparticles were characterised in terms of median volume diameter by laser light scattering. In short, an amount of dry powder (15 mg) was dispersed in 15 mL of 2-propanol and sonicated for 5 min. Measurements were performed with a SprayTec^®^ (Malvern, UK) and data are expressed as 50% (D_v_50) of aerosol droplets. The analyses were performed three times with an obscuration threshold of 10% [13].

#### 2.3.4. Density

A helium pycnometer (Micromeritics AccuPyc 1330, Aachen, Germany) was used to determine microparticle real density (g/cm^3^; *n* = 3). Tap density (g/cm^3^) was determined using a tap density tester (*n* = 3; 30 tapping/min, Densipro 250410, Deyman, Santiago de Compostela, Spain).

### 2.4. Drug Association Efficiency and Loading

In order to determine the drug content, the dry powder (20 mg) was solubilised in HCl 0.1 M (10 mL) under magnetic stirring for 20 min. Then, the solution was filtered (0.45 µm, RC, Sartorius, Concord, CA, USA), and a sample was analysed by high-performance liquid chromatography (HPLC—Agilent 1100 series, Concord, Germany). A LiChrospher^®^ 100 RP-18 (4.6 µm) column of 4 mm i.d. × 250 mm length with a security guard cartridge was used, and detection was performed by a diode array detector set at a wavelength of 275 nm. For the analysis, the mobile phase consisted of phosphate buffer 20 mM pH = 7 (A) and acetonitrile (B), flowing at a rate of 1.0 mL/min. The elution was conducted with a gradient starting with A/B = 95%/5% (0–5 min), which further reached a 30%/70% A/B ratio (5–8 min), which was kept for 19 min. Under these conditions, retention times of INH and RFB were 5 min and 20 min, respectively. Calibration curves (10–400 mg/mL) were plotted using INH and RFB standard solutions (HCl 0.1M). Drug association efficiency and microparticle loading capacity were calculated (*n* = 3) by the following equations [14,15]:

AE (%) = (Real drug content/Theoretical drug content) × 100(1)

LC (%) = (Real amount of drug/Weight of MP) × 100(2)

### 2.5. In Vitro Drug Deposition

The in vitro aerosolisation of the dry powder was evaluated using the Andersen cascade impactor (ACI, Copley Scientific Ltd., Nottingham, UK). The used methodology respected the USP38 guidelines for dry powder inhalers (Apparatus 1, United States Pharmacopoeia, Chapter 601). ACI separates particles according to their aerodynamic diameter, and it was assembled using the appropriate adaptor kit for the 60 L/min air flow test. Cut-offs of the stages from −1 to 6 are the following: 8.60, 6.50, 4.40, 3.20, 1.90, 1.20, 0.55 and 0.26 µm. A glass fiber filter (Whatman, Milano, Italy) was placed right below stage six in order to collect particles with a diameter lower than that of the stage six cut-off. Collection plates were coated with 1% (*v/v*) Tween^®^ 20 in ethanol to prevent particle bounce.

A powder amount of 30 mg was loaded into a size three HPMC capsule (Quali-V-I, Qualicaps, Madrid, Spain) and aerosolised using an RS01 device (IFR = 0.033 kPa^0.5^/LPM, Plastiape, Lecco, Italy). The content of three capsules was discharged for each experiment, and the experiments were performed in triplicate.

The flow rate that was used during each test was adjusted at 60 L/min with a critical flow controller TPK (Copley Scientific, Nottingham, UK) in order to produce a pressure drop of 4 kPa across the inhaler. The flow rate corresponding to these pressures was measured before each experiment using a DFM 2000 Flow Meter (Copley Scientific, Nottingham, UK). The test duration time was adjusted at 4 s, so that a volume of 4 L of air was drawn through each inhaler during each test.

A mixture of water/acetonitrile (50/50, *v/v*) was used to rinse off the powder from the apparatus. Samples were then sonicated for 5 min, filtered (0.45 µm, RC, Sartorius, Concord, CA, USA), and analysed by HPLC (Agilent 1200 series, Waldbronn, Germany), following the analytical protocol previously described. 

The measurement of the INH and RFB that was deposited in the impactor allowed the calculation of deposition parameters. Mass median aerodynamic diameter (MMAD) was determined by plotting the cumulative percentage of mass less than the stated aerodynamic diameter for each stage on a probability scale versus the aerodynamic diameter of the stage on a logarithmic scale. The mass of drug particles with size <5 µm (calculated from log-probability plots) was defined as a fine particle dose (FPD). Yet, the amount of drug leaving the device and reaching the impactor was considered as the emitted dose (ED). Subsequently, the fine particle fraction (FPF) was calculated as the percentage ratio between FPD and ED. Finally, the metered dose (MD) was accounted as the mass of drug recovered and quantified by HPLC, and was calculated by summing the drug recovered from the inhaler (device and capsule) and the impactor (induction port, stages −1 to 6 and F).

### 2.6. Drug Release Profiles

The in vitro drug release studies were carried out in PBS (pH 7.4) and in buffer solution at pH 5 (citric acid ~0.096 M, sodium hydroxide ~0.20 M, Sigma-Aldrich, Munich, Germany), both containing 1% (*v/v*) Tween^®^ 80. Assays were performed respecting sink conditions, as the maximum amount of drug was always below 30% of its maximum solubility [16]. The drug release rate was determined by incubating the dry powder (20 mg) with a release medium (10 mL) in a test tube, which was kept at 37 °C (Dry line; VWR, Tempe, AZ, USA) under mild shaking (100 rpm; Orbital Shaker OS 10, Biosan, Riga, Latvia). Then, at pre-established time intervals, aliquots (1 mL) were withdrawn, centrifuged (16,000× *g*, 15 min; Heraeus Fresco 17 Centrifuge, Thermo Scientific, Waltham, MA, USA), and filtered (0.45 µm). In the end, the drug content in the samples was quantified by HPLC (*n* = 3) by interpolation from calibration curves obtained with standard solutions of drugs diluted in the release media. 

### 2.7. In Vitro Biocompatibility Studies 

#### 2.7.1. Cell Culture

Human alveolar epithelium cells (A549) were purchased from the American Type Culture Collection (ATCC, Middlesex, UK). The cell line was cultured in DMEM supplemented with 10% (*v/v*) of FBS, 1% (*v/v*) l-glutamine solution 200 mM, 1% (*v/v*) non-essential amino acids, and 1% (*v/v*) penicillin/streptomycin. For the experiments, cells were used in passages 25–36.

THP-1 human monocytic cells were obtained from the Leibniz-Institute DSMZ (Braunschweig, Germany). The cell line was maintained in suspension at 0.2–0.8 × 10^6^ cells/mL in RPMI 1640 medium supplemented with 10% (*v/v*) FBS, 1% (*v/v*) l-glutamine, and 1% (*v/v*) penicillin/streptomycin. For the assays, THP-1 cells (0.35 × 10^6^ cells/mL) were differentiated to acquire macrophage phenotype (50 nM PMA, 48 h) before performing the cytotoxicity tests. Cells were used between passages 10–17.

In general, cell cultures were grown using 75 cm^2^ flasks in a humidified 5% CO_2_/95% atmospheric air incubator at 37 °C (HerAcell 150, Heraeus, Hanau, Germany).

#### 2.7.2. Determination of Metabolic Activity

The effect of FUC microparticles on cell viability was evaluated on A549 and macrophage-differentiated THP-1 cells by MTT assay. Briefly, A549 cells were seeded in 96-well plates (Orange Scientific, Braine-l’Alleud, Belgium) at a density of 1.0 × 10^4^ cells/well in complete medium (100 µL) and allowed to attach overnight at 37 °C in 5% CO_2_ atmosphere. After that, cells were exposed to test solutions for 3 and 24 h. After the exposure time, the medium was removed, and 30 µL of MTT (0.5 mg/mL in PBS, pH 7.4) were added in each well, followed by 2 h of incubation at 37 °C. Next, purple crystals were dissolved with DMSO (50 µL), and the absorbance was determined by spectrophotometry (Infinite M200, Tecan, Grödig, Austria) at 540 nm, subtracting the background absorbance (640 nm). 

Similarly, THP-1 cells were seeded in 96-well plates (0.35 × 10^6^ cells/well) in 100 µL of complete medium and differentiated as described before. After differentiation, the cell culture medium (CCM) was renewed for another 24 h, before performing the experiments. As for A549 cells, exposure times of 3 and 24 h were applied. After exposure, MTT solution (30 µL) was added in each well (no media removal was applied), and incubation was allowed for 2 h, after which formazan crystals were solubilised with 10% SDS in a 1:1 mixture of DMF. The absorbance was determined by spectrophotometry, as described above.

Overall, test solutions were previously prepared by dissolving the microparticles (unloaded and drug-loaded) in the proper CCM without FBS at three concentrations: 0.1, 0.5, and 1.0 mg/mL. INH and RFB were also tested as free drugs at concentrations equivalent to their theoretical loadings in microparticles, i.e., 0.01, 0.05 and 0.1 mg/mL for INH and 0.005, 0.025 and 0.05 mg/mL for RFB. 

CCM and 2% (*w*/*v*) SDS were used as positive and negative controls of cell viability, respectively. Cell viability of treated cells was expressed as a percentage of that observed for the positive control (CCM). The assay was performed at least three times, with six replicates at each concentration of test solutions.

#### 2.7.3. Evaluation of Cell Membrane Integrity

The integrity of cell membrane was assessed by the quantification of LDH release upon exposure to test samples. Both A549 (1.0 × 10^4^ cells/well) and macrophage-differentiated THP-1 cells (0.35 × 10^6^ cells/well) were exposed to sample solutions at a concentration of 1.0 mg/mL (corresponding to the maximum concentration used in MTT assays). Solutions of free INH (0.1 mg/mL) and RFB (0.05 mg/mL) were also tested. After 24 h of exposure, aliquots (100 µL) of cell supernatant samples were centrifuged (16,000× *g*, 5 min) and processed using a commercial LDH kit (Takara Bio, Tokyo, Japan). The concentration of LDH was measured by spectrophotometry (Infinite M200, Tecan, Grödig, Austria) at a wavelength of 490 nm with background correction at 690 nm. Cells incubated with CCM only were considered the negative control; those treated with Triton-X100 (10%) were used as the positive control, the latter being assumed as 100% of LDH release. Thus, released LDH values were expressed as a percentage of the positive control. All of the measurements were performed in triplicate.

### 2.8. Macrophage Activation by Microparticles

In order to evaluate the capability of microparticles to activate macrophage-like cells, differentiated THP-1 cells (0.350 × 10^6^ cells/well) were incubated with drug-loaded FUC microparticles. After 24 h of incubation, cell-free supernatants were collected and TNF-α and IL-8 quantified with Quantikine^®^ HS ELISA kits (R&D Systems, Minneapolis, MN, USA). The amount of each cytokine was expressed in pg/mL based on reference standard curves. Cytokines released from cells treated with FUC solution and LPS, and untreated cells were used as controls. The absorbance of samples was determined at 450 nm in a microplate reader and corrected for background absorbance at 540 nm.

### 2.9. Preliminary Evaluation of Microparticle Uptake by Macrophages

The uptake of FUC microparticles by macrophage-like cells was performed by flow cytometry (FacScalibur cell analyser, BD Biosciences, Erembodegem, Belgium) and involved the exposure of cells to fluorescently-labelled microparticles. The assay was performed on human macrophage-differentiated THP-1 cells and on rat alveolar macrophages (NR8383 cells). Macrophage-differentiated THP-1 (0.35 × 10^6^ cells/mL) and NR8383 (0.2 × 10^6^ cells/mL) cells were seeded in 35 mm-diameter dishes, containing 5 mL of the respective complete medium. Next, cells were maintained for 24 h at 37 °C to ensure the adhesion of 50%–75% of the original population. Then, media were removed, and fluorescent microparticles (50 and 200 µg/cm^2^) were aerosolised onto the macrophage layer using a Dry Powder Insufflator™ (Model DP-4, Penn-Century™, Wyndmoor, PA, USA). Cells unexposed to microparticles were considered as negative control. The phagocytic process was allowed for 2 h (incubation at 37 °C) and was stopped by the addition of a cold solution of PBS.3% FBS (5 mL, two applications). Then, cells were scraped, re-suspended in 3 mL of PBS.3% and centrifuged (1500 rpm, 2 min, room temperature, centrifuge MPW-223e, MedInstruments, Warsaw, Poland). Cells were washed with PBS.3% (5 mL) thrice and finally re-suspended in 1 mL of buffer for flow cytometry analysis (BD Biosciences FACSCalibur, Erembodegem, Belgium). For this purpose, side scatter light was used to distinguish cell viable population, whereas FSC-H and SSC-H channels were applied to measure the size and granularity of cells, respectively. A total of 10,000 events were counted within a gated region, and the data were presented as mean fluorescence (FL) intensity. The number of cells associated with fluorescence was considered the definition for uptake. The assay was replicated at least three times for each dose.

### 2.10. Determination of Minimum Inhibitory Concentration (MIC) 

#### 2.10.1. Culture of Mycobacteria

The in vitro efficacy of microparticles was evaluated against *Mycobacterium bovis* BCG (DSMZ 43990), provided, as a gift, by Centro de Estudos de Doenças Crónicas da Faculdade de Ciências Médicas da Universidade Nova de Lisboa (CEDOC/FCM-UNL). The stocks of mycobacteria were preserved and stored in −80 °C ultralow temperature freezers (U725 Innova New Brunswick Scientific, Edison, NJ, USA). Mycobacteria were cultivated in M7H9 broth, supplemented with 10% OADC and 0.05% of Tween^®^ 80. Mycobacteria was handled inside a laminar flow hood (Bio48 Faster, Cornaredo, Italy), respecting the guidance and safety requirements to prevent contamination. That includes autoclave sterilisation (Uniclave88, Sintra, Portugal) of infectious materials.

#### 2.10.2. MIC Measurements

The lowest concentration of free antibiotics required to inhibit mycobacteria growth was determined by MTT assay [17]. Firstly, stock solutions (1 mg/mL) of INH and RFB were prepared by dissolving the drugs in the M7H9 supplemented medium. RFB was previously solubilised in ethanol, and then diluted with M7H9 broth. Stock solutions were filtered (0.22-μm sterile syringe filter) and mixed at concentrations proportional to the drug mass ratio contained in the microparticles (FUC/INH/RFB = 10/1/0.5, *w*/*w*). The susceptibility of the *M. bovis* strain was then evaluated by incubating mycobacteria with a drug solution combining INH/RFB, followed by serial dilutions. 

Similarly, the growth inhibition of mycobacteria promoted by FUC/INH/RFB microparticles was also assessed. A solution of dry powder was prepared at 1 mg/mL and then diluted, based on the calculations, to meet the desired drug concentrations. Generally, 96-well flat-bottom microplates (Orange Scientific, Braine-l’Alleud, Belgium) were filled with test samples, according to the scheme displayed in Figure S1 (Supplementary Materials). Three bacterial suspensions were prepared, and the assays were conducted after achieving an optical density value (*OD*_600nm_) of approximately 0.2, as measured by spectrophotometry (Infinite M200, Tecan, Austria). For the experiment, solutions (180 µL) of free drugs or microparticles were added into the wells (column 4), making continuously serial two-fold dilutions with M7H9 supplemented broth (columns 5–11). Next, 20 µL of bacterial suspension were added into the respective well, completing the final volume of 200 μL per well. Wells filled only with M7H9 supplemented medium (column 2; 200 µL) were used as a negative control. Similarly, bacterial suspensions (20 µL) were introduced in wells (column 3) containing M7H9 medium (180 µL) in the absence of free drugs/microparticles, which were assumed as positive controls. The bacterial growth of each bacterial suspension—1, 2, and 3—was evaluated in the B–C, D–E, and F–G lines, respectively. Assays were performed in triplicate.

The outside lane of wells (a frame-like) were filled with sterile distilled water to avoid the evaporation of microplate content. The plates were covered with the lid, sealed with parafilm, and incubated at 37 °C (Binder, Tempe, AZ, USA) for seven days. After that time, 30 μL of MTT sterile solution was added to each well, followed by 1 h of incubation at 37 °C. Then, 50 μL of DMSO was added into the wells, resulting in a colour change from yellow to dark gold proportional to the growth of mycobacteria. The absorbance was measured by spectrophotometry (Infinite M200, Tecan, Austria) at 540 nm. The minimum inhibitory concentration was considered the one that inhibited mycobacteria growth by 95% to 100%.

### 2.11. Statistical Analysis

Statistical significance was determined with the student *t*-test and one-way analysis of variance (ANOVA) with the pairwise multiple comparison procedures (Holm-Sidak method). A *p*-value of less than 0.05 was considered significant. Analysis was run using Sigmaplot software (version 12.5, Systat Software Inc., London, UK). 

## 3. Results and Discussion

### 3.1. Preparation and Characterisation of Fucoidan Microparticles

Spray-dried FUC microparticles loaded with a combination of two first-line antitubercular drugs (INH and RFB) were produced with a yield around 81%, indicating the effectiveness of the process. Drugs were efficiently associated to microparticles, with INH registering 97% ± 4% and RFB 95% ± 4% association efficiency. In this manner, loading capacities reached 8.5% ± 0.4% (INH) and 4.1% ± 0.2% (RFB), which were close to the theoretical values. This approach of associating antitubercular drugs in a single formulation meets the recommendations of the WHO regarding the need to establish a combined therapy for TB [12]. The selected theoretical drug loadings (8.7% for INH and 4.4% for RFB) are similar to those reported in other works [18]. INH is present in a higher amount than RFB, because the latter is a more potent drug [19], and also has a more toxic profile, as demonstrated in Section 3.4. Moreover, fucoidan proportion was kept purposely high in order to favour macrophage internalisation [8].

The fact that both drugs were associated with similar efficiencies demonstrates that the process was independent of the aqueous solubility of the drugs (125 mg/mL for INH and 0.19 mg/mL for RFB) [20,21]. 

**Figure 2:**
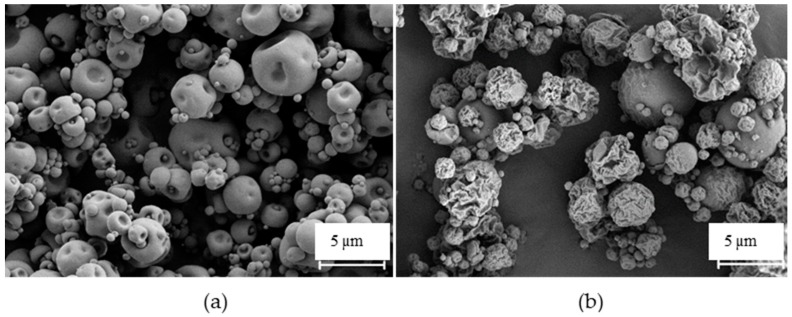
Scanning electron microphotographs of FUC-based microparticles: (**a**) unloaded microparticles and (**b**) FUC/INH/RFB microparticles. FUC: fucoidan, INH: isoniazid, RFB: rifabutin.

The morphological analysis performed with electronic microscopy revealed that the unloaded microparticles exhibited a slightly convoluted shape but smooth surface (Figure 2a). However, the incorporation of drugs in the microparticles produced important modifications on their morphology, which became more irregular and acquired corrugated surfaces (Figure 2b). In this case, the presence of RFB may have contributed to the morphological alterations perceived on the produced microparticles, as the observed surface wrinkles have been reported for other spray-dried microparticles loaded with RFB [22]. The wrinkled surface can also be thought of as a result of the drying process, in which the removal of ethanol from the microparticles’ surface occurs faster than water evaporation. The higher volatility of ethanol induces the formation of a primary shell that collapses as the water content in the core evaporates [23].

Drug-loaded microparticles were found to have a Feret’s diameter of 1.4 ± 0.8 µm; no significant differences were detected after drug incorporation (1.6 ± 0.8 µm for unloaded FUC microparticles). Additionally, volume diameter measurement by laser light scattering indicated median diameters (D_v_50) of 2.77 ± 0.03 µm for drug-loaded microparticles. The latter indicates that the produced microparticles present a suitable size for pulmonary deposition [24]. The particle size distribution reflects the productive efficiency of spray-drying to provide microparticles with favourable aerodynamic properties for inhalation purposes [25]. To the best of our knowledge, this is the first report describing the use of spray-drying to produce respirable FUC-based microparticles loaded with two antitubercular drugs.

Density further influences the properties of inhalable dry powders [26]. Thus, real and tap densities of FUC microparticles were determined, showing values around 1.742 ± 0.004 g/cm^3^ and 0.346 ± 0.019 g/cm^3^, respectively. These results are similar to others reported for spray-dried polysaccharide microparticles [27,28].

### 3.2. Aerodynamic Characterisation of Fucoidan Microparticles

The design of inhalable microparticles requires adequate flowability to promote lung deposition. In light of this, the in vitro aerosol performance was assessed in the ACI by using an RS01 dry powder inhaler. The obtained data are presented in Table 2.

The results demonstrated that more than 85% of the drugs were emitted from the device, indicating the suitability of FUC to be used as a matrix material in spray-dried inhalable microparticles. The adequate flowability is possibly a result of the wrinkled surfaces observed in the produced microparticles. Surface irregularities can reduce the cohesion forces between particles that lead to agglomeration, enhancing powder dispersibility, as well as improving the respirable fraction of the formulation [23,29]. This aspect is crucial when a lactose carrier is not included in the formulation, and spray-dried microparticles are aerosolised alone [16]. The FPF (≤5 µm) was around 50%, which represents the respirable fraction of microparticles with the potential ability to reach the respiratory zone. The results suggest as well that there was low cohesion among microparticles, which led to high deaggregation during aerosolisation and in agreement with the in vitro respirability usually exhibited by DPI formulation [24]. Additionally, the use of the RS01 device may have contributed to the maximisation of the performance, as it is described that the spinning capsule rotation provided by this inhaler is more efficient compared with the other capsule motion in the powder deaggregation [30,31].

**Figure 3:**
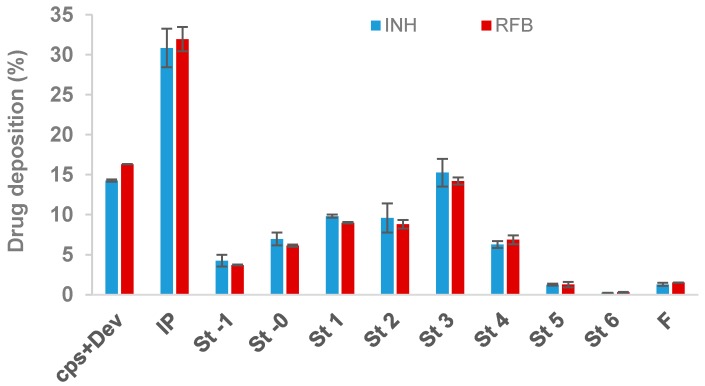
In vitro aerodynamic deposition of antitubercular drugs (INH and RFB) in the Andersen cascade impactor. Drugs were associated with spray-dried fucoidan microparticles. Values are mean ± SD, *n* = 3. Cps: capsule; Dev: inhaler device; IP: induction port; F: filter, INH: isoniazid; RFB: rifabutin St: stage.

Figure 3 illustrates the stage-by-stage deposition profiles of INH and RFB in the ACI after aerosolisation. The drug recovery varied between 82%–85%, being in accordance with the values established by the European Pharmacopeia [32]. According to drug mass deposition on ACI stages, the determined MMAD values were 3.9 µm (INH) and 3.6 µm (RFB), suggesting a suitable size for lung deposition. In fact, particles with an aerodynamic diameter within the range of 1–5 µm display a greater tendency for reaching the respiratory zone, and if with extrafine size (<2 µm), have more peripheral deposition within the area [33]. Moreover, they are in the adequate size range (1–6 µm) that allows phagocytosis by macrophage, the target cells [34].

The similarity between the profiles of both drugs demonstrates that INH and RFB were equally co-deposited on the several stages, thus supporting the decision of developing a delivery system with a combination of the two drugs. Furthermore, this indicates that the drugs have even distribution within the microparticles. In summary, the produced microparticles were shown to have adequate aerodynamic characteristics for the pulmonary delivery of antitubercular drugs with great propensity to reach the respiratory zone.

### 3.3. In Vitro Drug Release Profiles

**Figure 4:**
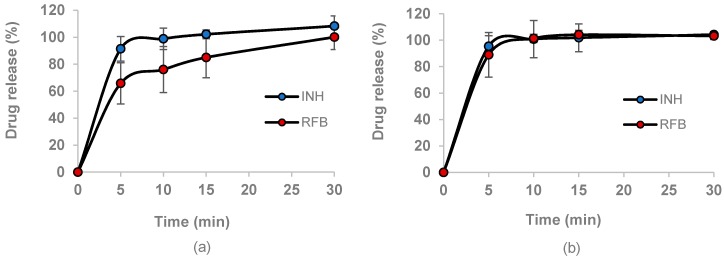
In vitro release profile of isoniazid (INH) and rifabutin (RFB) from FUC/INH/RFB (10/1/0.5, *w*/*w*) in (**a**) PBS pH 7.4-Tween 80^®^ and (**b**) acidic medium pH 5.0-Tween 80^®^. FUC: fucoidan; mean ± SD, *n* = 3.

The release of drugs was evaluated in PBS (pH 7.4) with the addition of 1% (*v/v*) Tween^®^ 80, resembling the lung lining fluid in terms of pH and the presence of surfactant [35,36]. The latter also enables the dissolution of RFB, which is sparingly soluble in aqueous media. Considering that alveolar macrophages are the target cells in this study, drug release studies were also conducted in more acidic medium (pH 5), simulating the phagolysosomal environment [37]. The obtained results are shown in Figure 4.

In general, the two drugs released rapidly from the microparticles, especially INH, which is completely available after 10 min, disregard the pH of the release medium (Figure 4a,b). The rapid release of this drug was expected, owing to its high solubility in water. However, the RFB release profile was also similar to that of INH, despite its lower solubility. At pH 5, RFB released exactly at the same rate as INH. In turn, at pH 7.4, the release was a little bit slower, although not to a statistically significant level. In this medium, approximately 75% of the RFB was released within 10 min, and 100% was released within 30 min. The faster release in acidic medium is possibly a consequence of a certain protonation of the drug, which increases its solubility. However, no significant difference was perceived at any time point, indicating that the release of the two drugs was not significantly influenced by pH. Other works also reported the rapid release of INH and RFB from polysaccharide microparticles [20,38].

The rapid release could be explained by two main factors: the surface irregularities, which increase the contact with the medium, and the high solubility of the polymeric matrix, i.e., FUC rapidly dissolves in the media, releasing the drugs. It should be stressed that these observations do not reflect in vivo occurrences, considering that a lower amount of liquid is present in the alveoli compared with that involved in the assays. It is well known that the alveolar epithelium is covered by a thin layer (0.01–0.1 µm) of lung lining fluid, and thus, microparticles are expected to be only partially in contact with this fluid, and not immersed in it [39,40]. Consequently, in vivo drug release will probably occur more slowly, allowing microparticle internalisation by macrophage cells before particle dissolution and complete drug release.

### 3.4. In Vitro Cytotoxicity

The cytotoxicity of FUC/INH/RFB microparticles was evaluated by performing two complementary tests: metabolic assay (MTT) and the LDH release assay, which assesses cell membrane integrity. In both cases, alveolar epithelial cells (A549) and macrophage-differentiated THP-1 cells were used. Drug-loaded microparticles were tested along with an unloaded formulation and free drugs, which were considered as controls. Free drugs were tested at concentrations corresponding to the respective theoretical loading in the microparticles.

#### 3.4.1. Metabolic Activity by MTT Assay

**Figure 5:**
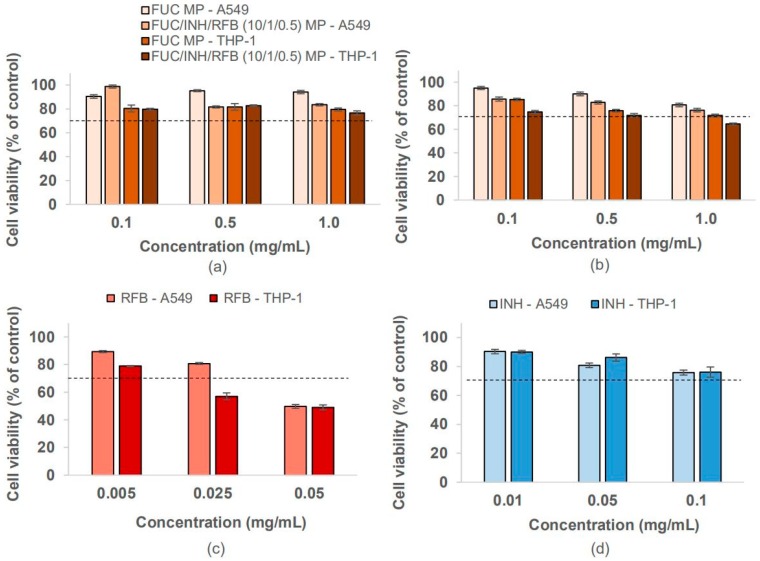
A549 and macrophage-differentiated THP-1 cell viability upon (**a**) 3 h and (**b**) 24 h of exposure to unloaded FUC and FUC/INH/RFB (10/1/0.5 *w*/*w*) microparticles; (**c**) 24 h exposure to RFB as a free drug; and (**d**) 24 h exposure to INH as a free drug. Cell viability was calculated as a percentage of positive control (untreated cells). Data represent mean ± SEM (*n* = 3, six replicates per experiment at each concentration). Dashed line indicates 70% cell viability. FUC: fucoidan; INH: isoniazid; MP: microparticles; RFB: rifabutin.

The MTT assay was performed upon 3 and 24 h of exposure, and revealed that the viability of the A549 cells exposed to the produced microparticles, in all of the tested conditions, remained above 70%, the threshold below which it is considered the occurrence of cytotoxic effect according to ISO [41]. In fact, the exposure of A549 cells to FUC/INH/RFB microparticles induced mild effects on cell viability; meanwhile, no significant differences were observed in terms of time and microparticle concentrations (Figure 5a,b lighter colours). However, macrophage-differentiated THP-1 cells were slightly more sensitive to the contact with FUC/INH/RFB microparticles after long-time exposure. At 24 h, cell viability decreased to 65% upon exposure to the highest dose of microparticles (1.0 mg/mL) (Figure 5b, darker colours). Comparatively, A549 cells showed 76% viability in the same conditions (*p* < 0.05). Concerning macrophage-like THP-1 cells, no significant time-dependent or dose-dependent effects were observed.

The mild toxicity observed in THP-1 cells is clearly due to the RFB content, as was already described in a recent work of our group [42]. Figure 5c,d depicts the effects of the free drugs after 24 h. While INH induced cell viabilities between 75%–90% in both cell lines, thus not exhibiting a cytotoxic behaviour, RFB generated different responses. In fact, RFB decreased cell viability to around 50% in both cell types when tested at the highest concentration (0.05 mg/mL), while the viability of differentiated THP-1 cells also decreased to 57% at the intermediate concentration (0.025 mg/mL, Figure 5c). These observations demonstrated the drug toxicity, which has been reported in vivo [43,44], and indicated that the toxic effect is due to RFB. In order to further confirm this, drug-loaded microparticles were produced with lower amounts of RFB (FUC/INH/RFB = 10/1/0.2, *w*/*w*) to be purposely tested regarding cytotoxicity. It was verified that this decrease in RFB content resulted in the viability of differentiated THP-1 cells over 70% in all of the tested concentrations (data not shown). Free drugs were also tested for 3 h (data not shown). INH induced the viability of 89%–99% in all of the cases. In turn, RFB decreased viability to 68% (A549 cells) and 56% (differentiated THP-1 cells) at the highest tested concentration (0.05 mg/mL). It should be stressed that at the same time point (3 h), viability remained at 84% (A549) and 77% (differentiated THP-1 cells) when cells were exposed to FUC/INH/RFB microparticles (1.0 mg/mL), as shown in Figure 5a. In this way, it is suggested that the microencapsulation had a beneficial effect on RFB cytotoxicity (*p* < 0.05). Unloaded FUC microparticles were also tested as control in both cell lines (Figure 5a,b), resulting in a cell viability above 80% in all of the cases, thus evidencing the absence of detrimental effects of the polysaccharide under the tested conditions.

Overall, it can be considered that both A549 and macrophage-differentiated cells tolerated the exposure to fucoidan-based microparticles well. A single exception was registered in macrophage-like THP-1 cells when exposed to 1.0 mg/mL microparticles for 24 h, with the toxicity being attributed to the RFB content. However, it should be highlighted that this dose is much higher than the one that was expected to occur in vivo, as after the delivery, the dry powder will be distributed through a large surface. Therefore, the effects will probably be more similar to those of the lower doses (0.1 and 0.5 mg/mL), which were concentrations where no cytotoxicity was perceived. 

#### 3.4.2. Cell Membrane Integrity

**Figure 6:**
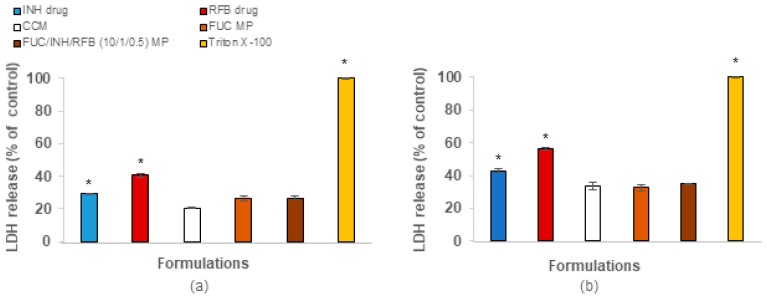
Release of lactate dehydrogenase (LDH) from (**a**) A549 cells and (**b**) macrophage-differentiated THP-1 cells exposed to fucoidan-based microparticles (1.0 mg/mL), free rifabutin (RFB, 0.05 mg/mL), and free isoniazid (INH, 0.1 mg/mL). Cell culture medium (CCM) and Triton X-100 were used as negative and positive controls, respectively. The released LDH calculated was based on 100% assumed for positive control. Data represent mean ± SEM (*n* = 3, six replicates per experiment at each concentration). * *p* < 0.05 compared to CCM.

As a complement to the MTT assay, the amount of the cytoplasmic enzyme LDH released after cell exposure (24 h) to microparticles (1.0 mg/mL) and free drugs was determined (Figure 6). The results essentially confirm those of MTT, with free RFB having a more intense effect on the release of LDH. The incubation with CCM induced 21% of LDH release in A549 cells (Figure 6a) and 34% in macrophage-differentiated THP-1 cells (Figure 6b). The exposure to free RFB significantly increased these values to 42% and 57%, respectively (*p* < 0.05). Despite RFB cytotoxicity, FUC/INH/RFB microparticles generated similar LDH release compared with CCM in both cell lines, which reinforces the beneficial effect of microencapsulation regarding RFB cytotoxicity. 

Curiously, free INH also induced the release of a significantly higher amount of LDH compared with the control CCM (29% for A549 cells and 43% for macrophage-differentiated THP-1 cells, *p* < 0.05). However, microencapsulation reverted the toxicological effect (*p* < 0.05). Unloaded FUC microparticles also evidenced an absence of effect on LDH release.

The overall evaluation of in vitro cytotoxicity indicates a very acceptable toxicological profile of FUC-based microparticles. Nevertheless, it is considered beneficial to widen the toxicological studies of these microparticles to certify their use for the proposed application.

### 3.5. Macrophage Activation

The ability of FUC/INH/RFB microparticles to induce macrophage activation was assessed by determining the amount of TNF-α and IL-8 secreted by macrophage-like THP-1 cells upon contact with the formulation. The referred cytokines are two pro-inflammatory molecules released by human alveolar macrophages upon infection with M. tuberculosis [45,46]. The synthesis of pro-inflammatory cytokines such as TNF-α and IL-8 by macrophages contributes to the effective control of the proliferation and dissemination of pathogens [47]. The amount of secreted cytokines was compared with the levels produced upon stimulation with LPS (positive control) and untreated cells (negative control). 

**Figure 7:**
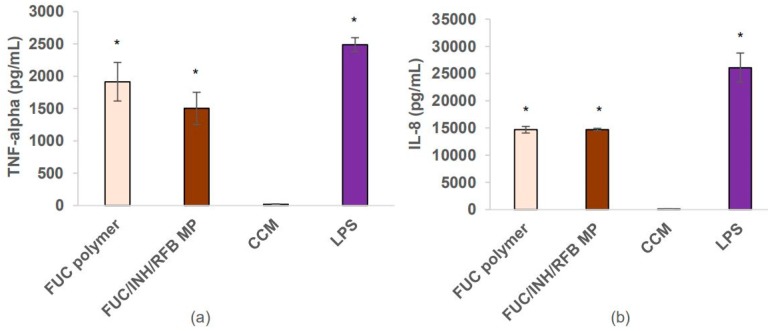
Assessment of (**a**) TNF-α and (**b**) IL-8 secretion induced by FUC/INH/RFB microparticles and FUC as the raw material. Lipopolysaccharide (LPS) and cell culture medium (CCM) were used as controls. FUC: fucoidan; INH: isoniazid; RFB: rifabutin. Data represent mean ± SEM (*n* = 3). * *p* < 0.05 compared to CCM.

Figure 7 depicts the obtained results. The contact with drug-loaded FUC microparticles induced TNF-α concentrations of 1.5 × 10^3^ pg/mL (Figure 7a), which did not differ statistically from the value registered upon LPS stimulation, although the nominal value was higher in that case (2.5 × 10^3^ pg/mL). This observation is in agreement with a recent report showing that that FUC induces TNF-α secretion from macrophage-differentiated THP-1 cells [48]. FUC was also tested as a solution, which gives an indication of the effect of the polymer itself. It was shown to induce the production of TNF-α (1.9 × 10^3^ pg/mL), which is not statistically different from the effect of microparticles. Importantly, when comparing with CCM, the induced TNF-α production was higher for both FUC microparticles and FUC solution (*p* < 0.05). 

Similar effects were observed regarding the production of IL-8 (Figure 7b). Although in this case, LPS generated a significantly higher amount of cytokine (26 × 10^3^ pg/mL, *p* < 0.05), drug-loaded FUC microparticles also revealed an ability to induce its secretion, which reached 14.7 × 10^3^ pg/mL, more than half of the amount corresponding to LPS. Moreover, IL-8 secretion stimulated by the produced microparticles and raw material were much higher than that of the control CCM (*p* < 0.05).

Overall, no significant differences were observed in the production of each interleukin upon stimulation by FUC/INH/RFB microparticles and the raw material FUC. In this way, the results suggest that FUC is the agent responsible for the observed activation of macrophage-like cells. Actually, the immune modulatory activity of FUC has been already reported, and is mediated through the regulation of immune cells, including macrophages [49,50]. Although FUC has been shown to induce the production of TNF-α [11,48] and IL-8 [51] from macrophages and other immune cells [9], the polymer seems to be endowed with anti-inflammatory activity as well. In truth, the mechanism of action of FUC as a bioactive agent is yet to be unveiled [52,53], and this is an area worth researching.

### 3.6. Preliminary Evaluation of Microparticle Uptake by Macrophage-Like Cells

**Figure 8:**
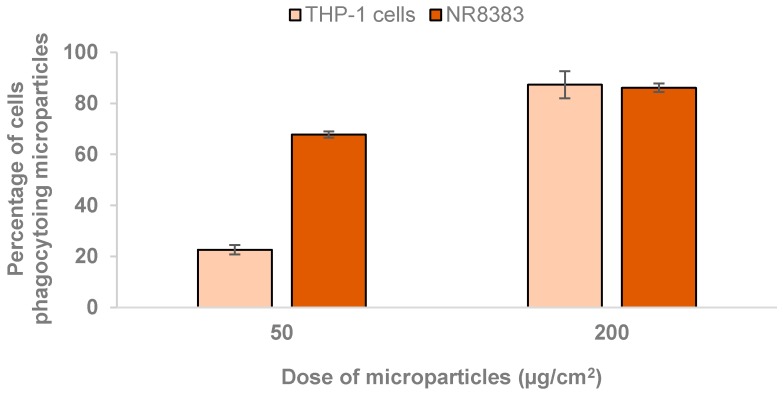
Uptake of fluorescently-labelled fucoidan (FUC) microparticles by macrophage-differentiated THP-1 cells and NR8383 cells exposure to 50 µg/cm^2^ and 200 µg/cm^2^, for a period of 2 h. Results are expressed as mean ± SEM (*n* ≥ 3).

Taking into consideration the intended application of the developed microparticles, the ability of macrophage cells to internalise the carriers was assessed by flow cytometry. Macrophage-differentiated THP-1 cells and rat alveolar macrophages NR8383 were exposed to two different doses of microparticles. As depicted in Figure 8, the percentage of THP-1 cells taking up FUC microparticles were around 23% (50 µg/cm^2^) and 87% (200 µg/cm^2^), demonstrating a dose-dependent uptake (*p* < 0.05). Similarly, the cellular uptake of carriers by rat macrophages varied between 68%–86% as the concentration raised from 50 to 200 µg/cm^2^, also revealing a dose-dependent effect (*p* < 0.05). Significant differences (*p* < 0.05) were observed between the uptake by both cell lines, particularly at the lowest tested dose (50 µg/cm^2^). In this case, 23% and 68% of FUC microparticles were taken up by THP-1 cells and NR8383 cells, respectively. Therefore, the obtained results are a preliminary demonstration of the macrophage’s ability to uptake FUC microparticles at a considerable level, depending on the dose. Considering that FUC exhibits in its structure of chemical motifs (sulphate groups and sugar units) that can be recognised by macrophage surface receptors [8], a more accurate determination of preferential macrophage phagocytosis would be provided by comparing the uptake of FUC microparticles with a material devoid of recognisable moieties. Additionally, complementing cytometry data with confocal microscopy images would be beneficial in corroborating the data obtained so far.

### 3.7. Determination of Minimum Inhibitory Concentration

In order to measure the minimum inhibitory concentration of the produced systems, *M. bovis* cells were treated with free drugs and FUC/INH/RFB microparticles. The viability of mycobacteria upon exposure to tested samples was calculated as a percentage of bacterial culture (control), which was considered as 100% of bacterial growth.

The MIC value determined for INH as free drug was 0.125 µg/mL, which is in the value range reported in the literature [54,55]. Comparatively, free RFB at a concentration of 0.004 µg/mL was sufficient to inhibit the growth of *M. bovis*, suggesting that RFB is a stronger antimycobacterial agent than INH, probably due to the higher lipophilicity that facilitates its internalisation through the cell membrane [56,57]. The literature reports variable MIC values of RFB, depending on the strain of *M. bovis* and on the method of susceptibility testing [58,59]. By combining both INH and RFB as free drugs in a single solution, the determined MIC values were 0.008 µg/mL (INH) and 0.004 µg/mL (RFB). It is worth noting that the MIC of INH reduced from 0.125 to 0.008 µg/mL when combined with RFB. Differently, either alone or in combination, RFB displayed the same MIC value (0.004 µg/mL). This indicates that the in vitro susceptibility of mycobacteria to INH is potentiated when the two antitubercular drugs were applied together. 

By exposing *M. bovis* to 0.08 µg/mL of FUC/INH/RFB microparticles, the viability of mycobacteria decreased to the minimum level, therefore being the referred concentration of the MIC value of the produced systems. It is important to highlight that, at this microparticle concentration, the drug content, considering their respective association efficiency, is approximately 0.008 µg/mL (INH) and 0.004 µg/mL (RFB). These concentrations correspond to the MIC values determined for the two drugs when tested in combination (as free drugs). Therefore, the inhibition effect on the growth of *M. bovis* was very similar when comparing drug-loaded FUC microparticles and the mixed solution of INH/RFB as free drugs. This observation indicates that the microencapsulation process had no effect on the antibacterial activity of the drugs. In summary, an in vitro susceptibility of *M. bovis* has been observed towards drug-loaded FUC microparticles with considerable growth inhibition, as expected.

## 4. Conclusions

Inhalable dry powders based on FUC were produced to associate two first-line antitubercular drugs (INH and RFB) in a single formulation. Spray-dried microparticles were produced with high drug association efficiencies (>95%) and displayed MMADs between 3.6 and 3.9 µm, and a FPF around 45%–50%, thus evidencing the suitable aerodynamic properties for pulmonary delivery with great potential to reach the respiratory zone. FUC-based microparticles had no effect on the cell viability of alveolar epithelial cells (A549), although a slight reduction in the viability of macrophage-differentiated THP-1 cells (65% viable cells) was observed when exposed for 24 h to concentrations as high as 1.0 mg/mL. However, this dose is expected to largely overpass that to be observed in vivo. The produced microparticles were further demonstrated to be captured by macrophage-like cells (23%–87% uptake) in a dose-dependent manner. Moreover, they were able to induce macrophage activation by potentiating the secretion of cytokines. Furthermore, drug-loaded microparticles showed potential activity against a strain of mycobacteria (95% growth inhibition). According to the obtained data, the proposed delivery carrier, combining two antitubercular drugs in a single formulation, is a promising tool for the inhalable treatment of pulmonary TB. Nevertheless, unveiling the effect of a long-term administration of FUC microparticles in vivo, as well as the in vivo antimicrobial efficacy, are very relevant aspects to address in the future.

## Figures and Tables

**Table 1:** Operating parameters of the spray-drying process.

Microparticles	Inlet T (°C)	Aspirator (%)	Feed rate (mL/min)
Unloaded FUC	125 ± 1	80	2.0
FUC/INH/RFB	145 ± 1	85	1.0

FUC: fucoidan; INH: isoniazid; RFB: rifabutin.

**Table 2:** Aerodynamic parameters of fucoidan microparticles loaded with isoniazid (INH) and rifabutin (RFB) in the combined formulation. Loaded amount of powder in the capsule was 30 mg, corresponding to 2.6 mg of INH and 1.4 mg of RFB, according to the drug content of formulation (*n* = 3, mean ± SD).

Drug	Metered Dose (mg)	Emitted Dose (mg)	MMAD (µm)	FPD < 5 µm (mg)	FPF < 5 µm (%)
INH	1.91 ± 0.26	1.64 ± 0.23	3.90 ± 0.01	0.82 ± 0.02	50.2 ± 2.4
RFB	1.29 ± 0.03	1.10 ± 0.02	3.64 ± 0.32	0.53 ± 0.01	45.4 ± 1.4

FPD: fine particle dose; FPF: fine particle fraction; MMAD: mass median aerodynamic diameter.

## Supplementary Materials

The following are available online at http://www.mdpi.com/2073-4360/10/6/636/s1, Figure S1: Scheme of the 96-well microplate showing columns 4–11 filled with solutions of free drugs or microparticles serially diluted with M7H9 broth, containing mycobacteria in triplicate: lines B–C (suspension 1), lines D–E (suspension 2) and lines F–G (suspension 3). Contents of column 2 (only M7H9 medium) and column 3 (bacterial suspensions in broth) were considered negative and positive control, respectively.
