# Supplementary material for: Inhalable Fucoidan Microparticles Combining Two Antitubercular Drugs with Potential Application in Pulmonary Tuberculosis Therapy
Source: Polymers (Basel). 2018 Jun 8;10(6):636. doi: 10.3390/polym10060636 (PMC6403622; doi:10.3390/polym10060636)
Supplement: Supplementary file 1 [file polymers-10-00636-s001.pdf]

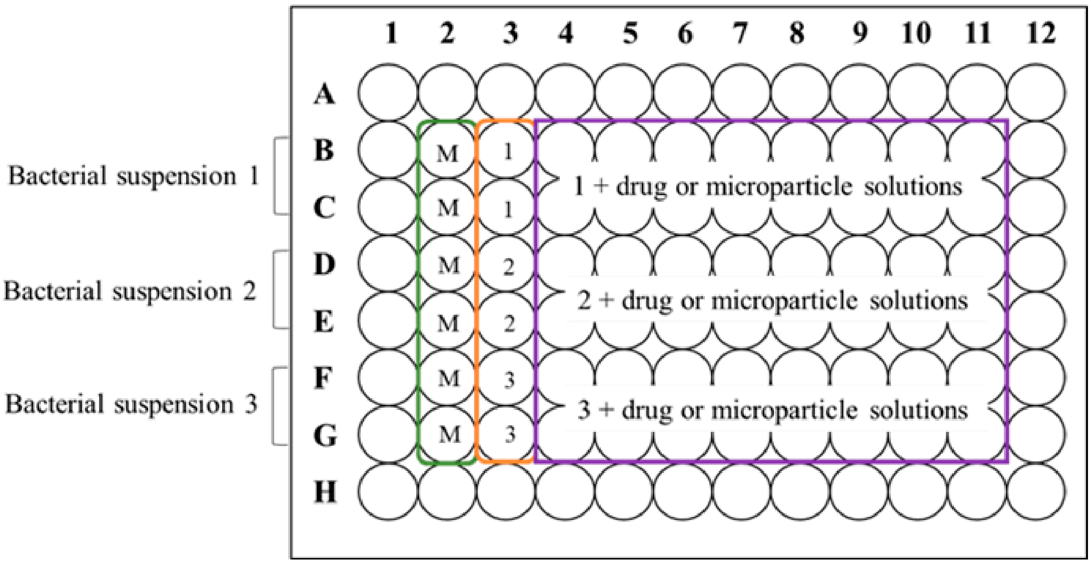

Figure S1. Scheme of the 96-well microplate showing columns 4-11 filled with solutions of free drugs or microparticles serially diluted with M7H9 broth, containing mycobacteria in triplicate: lines B-C (suspension 1), lines D-E (suspension 2) and lines F-G (suspension 3). Contents of column 2 (only M7H9 medium) and column 3 (bacterial suspensions in broth) were considered negative and positive control, respectively.
